# Aerobic exercise training enhances cerebrovascular pulsatility response to acute aerobic exercise in older adults

**DOI:** 10.14814/phy2.13681

**Published:** 2018-04-19

**Authors:** Nobuhiko Akazawa, Koichiro Tanahashi, Keisei Kosaki, Song‐Gyu Ra, Tomoko Matsubara, Youngju Choi, Asako Zempo‐Miyaki, Seiji Maeda

**Affiliations:** ^1^ Faculty of Health and Sport Sciences University of Tsukuba Tsukuba Japan; ^2^ Japan Institute of Sport Sciences Kita‐ku Tokyo Japan; ^3^ Graduate School of Comprehensive Human Sciences University of Tsukuba Tsukuba Japan; ^4^ Japanese Society for the Promotion of Science Chiyoda‐ku Tokyo Japan; ^5^ Faculty of Sports and Health Science Fukuoka University Fukuoka Japan; ^6^ Faculty of Medicine University of Tsukuba Tsukuba Japan; ^7^ Faculty of Health and Sport Sciences Ryutsu Keizai University Ibaraki Japan

**Keywords:** Aging, arterial stiffness, cerebral pulsatility, exercise training

## Abstract

The brain's low resistance ensures a robust blood flow throughout systole and diastole and is susceptible to flow pulsatility. Increased cerebral pulsatility contributes to the progression of cerebrovascular disease. Although aerobic exercise affects vascular function, little is known about the effect of exercise on the cerebral pulsatility index in older adults. The aim of this study was to investigate the effect of exercise training on the post‐exercise cerebral pulsatility response in older adults. Ten healthy older adults participated in a 12‐week exercise training intervention. Before and after the intervention, we measured the pulsatility index of the middle cerebral artery by means of transcranial Doppler method at baseline and following a cycling exercise bout performed at an intensity corresponding to the ventilatory threshold. Before exercise training, there was no significant change in the cerebral pulsatility response to an acute bout of cycling exercise. However, after the intervention, cerebral pulsatility decreased significantly following 30 min of an acute cycling exercise (*P* < 0.05). This study demonstrated that cerebral pulsatility index did not change following an acute bout of cycling exercise at an intensity corresponding to ventilatory threshold, but that, after 12 weeks of exercise training, cerebral pulsatility index was reduced at 30 min after a single bout of cycling exercise. These results suggest that long‐term aerobic exercise training may enhance the post‐exercise reduction in pulsatility index in older adults.

## Introduction

The central arteries have the ability to cushion oscillations in blood pressure and flow. The arterial cushioning function dampens most of the flow pulsatility from the heart. Stiffened arteries, however, have a decreased ability to cushion the pulsatility of blood flow, which contributes to high pulsation into the peripheral organs such as the brain and kidney (O'Rourke and Hashimoto 2007; Adji et al. 2011). The brain is a high flow and low resistance organ, and receives a relatively high blood flow to maintain its metabolism. As the heart pulsates due to blood afflux and efflux, the brain is passively perfused with a pulsatile flow throughout cardiac systole and diastole. The exposure of small vessels to highly pulsation results in microvascular damage (O'Rourke and Safar 2005). The pulsatility index (PI) of the middle cerebral artery, as measured by transcranial Doppler, correlates with the aortic pulse wave velocity, which is an index of central arterial stiffness (Kidwell et al. 2001; Webb et al. 2012; Tarumi et al. 2014). Cerebrovascular flow pulsatility increases with advancing age (Bakker et al. 2004). Previous studies have been demonstrated that an increase in the cerebral PI is associated with cerebral infarction in patient with diabetes mellitus and Alzheimer's dementia (Lee et al. 2000; Altmann et al. 2016). Management of the age‐related increase in cerebrovascular pulsatility is therefore important. Regular exercise is clinically efficacious in preventing and treating vascular aging (Ainslie et al. 2008; Seals et al. 2009), but the effects of exercise on cerebral PI in older adults have not been fully investigated.

Aerobic exercise (e.g., cycling exercise) increases cerebral blood flow and pulsatility (Jørgensen et al. 1992; Ogoh and Ainslie 2009). The increased cerebral pulsatility is considered to be due to increased arterial stiffness in response to a neurogenic mechanism (Heckmann et al. 2000). Young men exhibit a post‐exercise decrease in arterial stiffness (Kingwell et al. 1997), but this post‐exercise decrease is not found in older people. However, Maeda et al. (2008) reported that in older adults who followed an exercise training program, arterial stiffness did decrease after an acute bout of exercise. Thus, it is possible that aerobic exercise training enhances central and cerebral pulsatility responses to acute exercise. However, to the best of our knowledge, no previous study has investigated that the effects of exercise training on the post‐exercise response of cerebral hemodynamics in older adults.

This study aimed to determine the effect of exercise training on the post‐exercise cerebral hemodynamic response. It was hypothesized that exercise training would enhance the post‐exercise reduction in cerebral pulsatility. We investigated the responsiveness of cerebral hemodynamics following a single bout of aerobic cycling before and after 12 weeks of aerobic exercise training in older adults.

## Material and Methods

### Subjects

Ten healthy older adults (52–66 years old; two men, eight women) participated in the study. The subjects were nonsmokers, nonobese, and had no cardiovascular or cerebrovascular disease, as assessed by medical history. None of them participated in regular exercise and had not participated in another clinical study within the past year. All women were postmenopausal. None of the subjects was taking hormone replacement therapy or medication affecting the cardiovascular system. All potential risks associated with the study were explained to the participants, and they gave written informed consent for participation in the study. All procedures were reviewed and approved by the ethical committee of the University of Tsukuba.

### Experimental protocol

All 10 subjects completed a 12‐week aerobic exercise training. Before and after the intervention, each subject performed an acute bout of exercise test that consisted of a 30‐min bout of aerobic cycling at an intensity corresponding to the individual's ventilatory threshold (VT). All participants were at least 3 h postprandial and had not consume caffeine or alcohol for 12 h or strenuous exercise for 24 h, prior to the exercise test. We measured arterial stiffness, cerebral hemodynamics, blood pressure, and heart rate (HR) before and 30 and 60 min after the acute exercise. At least 2 days before the acute exercise test, a maximal exercise test and blood chemistry measurements were conducted after an overnight fast. All measurements were performed at a constant room temperature (24–26°C).

### Aerobic exercise training regimen

Participants underwent aerobic exercise training 4–6 days per week (3 supervised sessions and 1–3 additional home‐based sessions) for a total of 12 weeks based on the our previous study (Akazawa et al. 2015). Initially, participants performed cycling or walking sessions for 30 min/day at a relatively low intensity (60% of their individually determined HR_peak_, determined by maximal exercise test). As their exercise tolerance improved, all participants increased the exercise intensity (65% in weeks 1–2, 70% in weeks 3–5, 75% in weeks 6–10, and 80% is weeks 11–12) and duration (35 min/day in 2 weeks 4, 40 min/day is weeks 5–8, and 45 min/day in weeks 9–12).

### Acute bout of exercise test

Before and after the exercise training program, subjects performed a steady‐state acute exercise test at their individual VT for 30 min using an electrically braked cycle ergometer (75XLIII; Combi Wellness, Tokyo, Japan). An investigator monitored the subject's HR and oxygen uptake during exercise and supervised each subject so that the target intensity could be determined at around 60 rpm not to be affected to work volume by speed of evolution (Akazawa et al. 2015).

### Measurements

#### Central arterial stiffness

The carotid *β*‐stiffness index was determined noninvasively using a combination of ultrasonography and simultaneous applanation tonometry of the common carotid artery. The common carotid artery was imaged using B‐mode ultrasound (EnVisor; Koninklijle Philips Electronics, Eindhoven, the Netherlands) equipped with a high‐resolution linear‐array transducer (7.5 Hz). Diameters were measured from the intima of the far wall to the media‐adventitia of the near wall. Pulsatile changes in the common carotid artery diameter were analyzed 1–2 cm proximal to the bifurcation. Carotid arterial pressure waveforms were obtained with arterial applanation tonometry incorporating an array of 15 micropiezoresistive transducers (Form PWV/ABI, Colin Medical Technology, Komaki, Japan), and were calibrated by equating the carotid mean arterial pressure and diastolic blood pressure to the brachial mean arterial pressure and diastolic blood pressure. The arterial lumen diameter at minimal diastolic relaxation and maximal systolic expansion of the vessel was measured at three points per frame and averaged. Each parameter was averaged over at least 10 continuous cardiac cycles. These images and waveforms were recorded on a computer for subsequent offline analysis (Matsubara et al. 2014). *β*‐stiffness was analyzed using the equation:

β−stiffness=ln(P1/P0)/[(D1−D0)/D0]

where P1 and P0 are the highest and lowest blood pressure, and D1 and D0 are the maximum and minimum diameters. The *β*‐stiffness index was calculated as an index of arterial stiffness adjusted for distending pressure (Hirai et al. 1989). Brachial blood pressure and heart rate were determined using an automated device (Form PWV/ABI, Colin Medical Technology, Komaki, Japan).

#### Cerebral hemodynamics

Cerebral blood flow velocities in the right middle cerebral arteries were measured using a 2‐MHz pulsed Doppler ultrasonography system (Ez‐Dop; DWL Electronische Systeme, Singen, Germany). Doppler signals were obtained by adjusting the position to obtain the maximum reflected signal. PI was calculated using the formula: PI = (peak systolic velocity – end‐diastolic velocity)/mean velocity (Kim et al. 2010). Cerebrovascular resistance index was calculated using the formula: cerebrovascular resistance = mean arterial blood pressure/mean velocity. PI and cerebrovascular resistance were measured over a 1‐min stable phase at baseline and 30 and 60 min after a single bout of exercise, and the averaged values of each parameter were calculated. Participants were instructed to breathe normally and to avoid holding their breath. End‐tidal carbon dioxide partial pressure (P_ET_CO_2_) was monitored using a metabolic cart equipped with a respiratory gas‐analyzer system (AE300S; Minato Medical Science, Tokyo, Japan).

#### Oxygen uptake at ventilatory threshold and peak oxygen uptake

VT and peak oxygen uptake ($\dot{V}$O_2peak_) were measured during the incremental cycle ergometer exercise test using online computer‐assisted circuit spirometry (AE300S; Minato Medical Science, Osaka, Japan). All subjects performed a symptom‐limited cycling exercise test (after a 2 min warm‐up at 20 W, followed by 10 W increases every min) to exhaustion or age‐predicted maximal HR. $\dot{V}$O_2peak_ was defined at the highest value recorded during the test. Each individual VT was calculated using a regression analysis of the slopes of carbon dioxide production, oxygen uptake, and the minute ventilation plot.

#### Blood chemistry

A blood sample was collected from the antecubital vein after an overnight fast. Serum total cholesterol, triglycerides, and plasma glucose were determined using standard enzymatic techniques with an automatic clinical chemistry analyzer (LABOSPECT 008; Hitachi, Tokyo, Japan).

### Statistical analyses

Data are expressed as means ± SD. The Shapiro‐Wilk test for normality indicated that carotid pulse pressure, carotid *β*‐stiffness, cerebral blood flow mean velocity, and PI were not normality distributed. Normality distributed data were analyzed using 2 (training: before and after training) × 3 (time: baseline, 30‐min post, and 60‐min post) factor repeated‐measures analysis of variance (ANOVA). In the case of a significant *F*‐value, a post hoc Bonferroni test was used to identify significant differences between mean values. The data which were not normality distributed were analyzed using the nonparametric Friedman test. When the results of this test were significant (*P* < 0.05), the Wilcoxon rank test for post‐hoc was used to compare changes from baseline, and differences before and after training. The false discovery rate was used to adjust *P* value of 0.017 for changes between three‐time points. Student's paired *t*‐tests were used in normality distributed data and the Wilcoxon rank test were used in non‐normality distributed data to whether there was any change in the variables measured at baseline between before and after training.

## Results

All subjects completed the 12‐week aerobic exercise training program. The average frequency of exercise training was 3.1 ± 0.3 days/week in supervised sessions and 1.9 ± 0.9 days/week in home‐based sessions (5.0 ± 1.0 days/week in total). After the training, total cholesterol was significantly decreased and individual oxygen uptake at VT and $\dot{V}$O_2peak_ were significantly increased (Table 1) (*P* < 0.05). There were no significant differences in body weight, triglyceride, or fasting blood glucose levels before and after the 12‐week exercise training (Table 1).

**Table 1:** Subjects characteristics before and after exercise training

	Before training	After training
Age (years)	62 ± 4	
Height (cm)	155 ± 6	
Weight (kg)	52 ± 6	52 ± 6
Total cholesterol (mg/dL)	226 ± 27	210 ± 23^a^
Triglyceride (mg/dL)	107 ± 123	113 ± 82
Blood glucose (mg/dL)	92 ± 7	90 ± 6
Work at VT (watts)	44 ± 26	67 ± 24^a^
Oxygen uptake at VT (mL/kg/min)	14 ± 3	17 ± 4^a^
Peak oxygen uptake (mL/kg/min)	24 ± 5	29 ± 6^a^

Data are means ± SD. VT, ventilatory threshold.

a *P* < 0.05 versus before training.

There were no differences in baseline brachial blood pressure, carotid blood pressure, and HR between before and after exercise training (Table 2). The *β*‐stiffness index at baseline significantly decreased after the 12‐week exercise training (*P* < 0.05). All participants completed a single 30‐min bout of aerobic cycling at the intensity of their individual VT before and after the training program. Brachial blood pressure and carotid blood pressure did not change following a single bout of exercise before and after the 12‐week training, and no significant interaction was found. HR and *β*‐stiffness indicated a main effect of time (acute bout of exercise). There was no significant change in HR before the training program. After the 12‐week training, HR was significantly elevated at 30‐min post‐exercise (*P* < 0.05), but no significant interaction was found. There was also no significant change in the *β*‐stiffness index before the training program. After the 12‐week training, *β*‐stiffness index was significantly decreased at 30 min post‐exercise (*P* < 0.05).

**Table 2:** Hemodynamics responses to a single bout exercise before and after training

		Base	P30	P60
Brachial systolic blood pressure (mmHg)	Before	117 ± 13	116 ± 8	116 ± 8
	After	117 ± 10	116 ± 11	119 ± 12
Brachial mean arterial blood pressure (mmHg)	Before	90 ± 10	92 ± 9	92 ± 9
	After	91 ± 8	89 ± 9	92 ± 10
Brachial diastolic blood pressure (mmHg)	Before	73 ± 10	73 ± 9	71 ± 7
	After	70 ± 8	71 ± 8	74 ± 9
Brachial pulse pressure (mmHg)	Before	44 ± 8	43 ± 7	45 ± 7
	After	47 ± 7	45 ± 8	46 ± 8
Carotid systolic blood pressure (mmHg)	Before	109 ± 5	107 ± 3	107 ± 3
	After	109 ± 4	106 ± 4	110 ± 4
Carotid pulse pressure (mmHg)	Before	37 ± 3	34 ± 2	36 ± 2
	After	39 ± 3	35 ± 2	36 ± 3
Heart rate (bpm)	Before	58 ± 7	62 ± 5	60 ± 5
	After	56 ± 6	60 ± 7^a^	60 ± 8
*β*‐stiffness (U)	Before	7.4 ± 3.9	6.6 ± 3.5	6.9 ± 4.0
	After	6.2 ± 3.0^b^	5.6 ± 2.8^a^^,^ b	6.2 ± 2.7

Data are means ± SD. Base, Baseline; P30, post 30 min; P60, post 60 min.

a *P* < 0.05 versus base.

b *P* < 0.05 versus before training.

Baseline peak systolic, end diastolic, mean blood flow velocity, cerebrovascular resistance, PI of the middle cerebral artery, and P_ET_CO_2_ did not change between before and after exercise training (Table 3). The cerebral velocities, vascular resistance, and P_ET_CO_2_ measures did not change following a single bout of exercise before and after the 12‐week training, and no significant main effect of time or training and no interaction effects were found. The absolute PI indicated no main effect of training but a main effect of time. There was also no significant change in PI before the training program. After the 12‐week training, PI was significantly decreased at 30‐min post‐exercise compared with the baseline (*P* < 0.05).

**Table 3:** Blood flow velocity of middle cerebral artery responses to a single bout exercise before and after training

		Base	P30	P60
Peal systolic velocity (cm/sec)	Before	88 ± 16	89 ± 17	85 ± 16
	After	87 ± 18	88 ± 17	86 ± 16
End diastolic velocity (cm/sec)	Before	40 ± 7	42 ± 8	40 ± 6
	After	38 ± 7	44 ± 8	41 ± 8
Mean velocity (cm/sec)	Before	59 ± 10	61 ± 11	59 ± 10
	After	58 ± 10	62 ± 11	59 ± 11
Vascular resistance (mmHg/cm/sec)	Before	1.58 ± 0.39	1.55 ± 0.32	1.60 ± 0.34
	After	1.62 ± 0.24	1.49 ± 0.34	1.60 ± 0.32
Pulsatility index (U)	Before	0.82 ± 0.11	0.77 ± 0.12	0.77 ± 0.11
	After	0.83 ± 0.10	0.72 ± 0.12^a^	0.78 ± 0.11
P_ET_CO_2_ (mmHg)	Before	36 ± 2	34 ± 3	35 ± 3
	After	36 ± 3	36 ± 2	35 ± 2

Data are means ± SD. P_ET_CO_2_, end‐tidal carbon dioxide partial pressure. Base, Baseline; P30, post 30 min; P60, post 60 min.

a *P* < 0.05 versus base.

**Figure 1:**
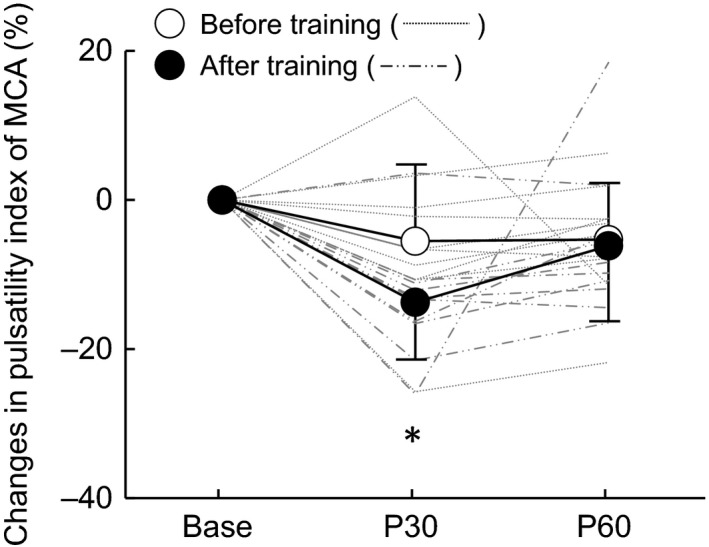
The response of the pulsatility index in the middle cerebral artery (MCA) before and after training. Thin lines represent individual changes. Base: baseline, P30: post 30 min acute exercise, P60: post 60 min acute exercise. *P < 0.05 versus Base.

Figure 1 shows the change in the PI in response to acute aerobic exercise before and after exercise training. The change in PI indicated no main effect of training, but a main effect of time. Before the exercise training, the PI did not change after the bout of acute aerobic exercise. After the training, however, the PI was significantly reduced 30 min after acute exercise (*P* < 0.05).

## Discussion

The main findings of the present investigation were as follows. First, before the 12‐week aerobic training intervention, arterial stiffness, cerebral blood flow velocities, and PI did not significantly change following a single bout of cycling at an intensity corresponding to VT. Second, after the intervention, arterial stiffness and cerebral PI decreased following 30 min of cycling exercise. These findings suggest that 12 weeks of aerobic exercise training reduced cerebral pulsatility and arterial stiffness after a single bout of exercise.

A unique feature of the brain is its perfusion throughout systole and diastole due to low resistance and high flow (O'Rourke and Safar 2005). The PI of the cerebral artery is thought to reflect downstream arterial resistance (Kidwell et al. 2001). A previous study reported that cerebral PI increased during exercise as a result of sympathetic nervous system activation (Heckmann et al. 2000). The present study investigated the post‐exercise cerebral pulsatility response in older adults. Before the intervention, an acute bout of cycling exercise did not decrease cerebral pulsatility, but after the 12‐week intervention, the blunted cerebral flow pulsatility response improved, as shown by the significant reduction in cerebral PI 30 min after a single acute bout of exercise. Therefore, exercise training may enhance the post‐exercise cerebrovascular pulsatility response.

Stiffened arteries lose their ability to cushion the pulsatile blood flow into peripheral organs (Adji et al. 2011). The pulse wave velocity of the central artery correlates with the PI of the middle cerebral artery in healthy adults (Tarumi et al. 2014). Kingwell et al. (1997) demonstrated that arterial stiffness decreased acutely after a single bout of aerobic exercise in young men. However, our group reported that, in older women, arterial stiffness did not change after an acute bout of exercise, but decreased after a period of exercise training (Maeda et al. 2008). Similarly, we recently reported that acute exercise decreased central aortic blood pressure in older adults who underwent a period of regular exercise training (Akazawa et al. 2015). Consistent with these studies, cerebral PI decreased with the reduction in arterial stiffness after the exercise training program. This study applied this notion to the post‐exercise cerebral blood flow response, and demonstrated that, after an exercise training intervention, a single bout of exercise acutely decreased central arterial stiffness, and consequently, cerebral flow pulsatility.

The effect of exercise training on cerebral hemodynamics at rest is controversial. A previous study (Tomoto et al. 2015) reported that cerebral blood flow velocity at rest did not change after a period of aerobic exercise training in young subjects, consistent with the results of the present study. In contrast, cross‐sectional studies have shown that exercise‐trained men with greater aerobic fitness have higher cerebral blood flow velocity than their sedentary peers (Ainslie et al. 2008; Bailey et al. 2013). Our study and others (Akazawa et al. 2012; Murrell et al. 2013) have demonstrated the possibility that aerobic exercise training increases cerebral blood flow velocity in older people. The discrepancy is probably due, in part, to the differences in exercise models, intervention periods, and sample sizes, as well as the time of day the measurements were taken because cerebral blood flow velocity fluctuates with the circadian rhythm (Conroy et al. 2005).

Cerebral PI at baseline also did not change after the 12‐week exercise training, although carotid arterial stiffness at baseline decreased. The training‐induced decrease in carotid arterial stiffness at rest did not correlated with the changes in PI and cerebrovascular resistance. Cerebral blood flow control is mediated by multiple regulatory factors such as perfusion pressure, metabolic activity, humoral factor, and autonomic nerve activity (Ainslie and Duffin 2009). A study in young men showed that cerebral blood flow velocity following acute exercise was maintained at baseline level despite post‐exercise hypotension and hypocapnia due to cerebral autoregulation (Willie et al. 2013). Cerebral arteriolar and capillary bed determined cerebral perfusion, cerebral autoregulation may help to control the pulsatility of the middle cerebral artery after a single bout of exercise or a period of exercise training. Taken together, the findings suggested that the relationships between central arterial stiffness and cerebral pulsatility at rest may be different from that after acute exercise and/or chronic exercise training. Further studies are warranted to investigate the effect of the regulation of cerebral hemodynamics to clarify the mechanism of the post‐exercise reduction in PI in older subjects.

We demonstrated that the reduction in PI disappeared within 60 min of exercise cessation. The implication that post‐exercise reduction in PI was transiently and not last for 60 min is unclear in this study. In a previous study, Kingwell et al. (1997) also reported that arterial stiffness decreased after 30 min and returned to resting level 60 min after exercise cessation in young men. The response of exercise‐induced decrease in arterial stiffness was likely to be mediated by post‐exercise sympathoinhibitation (Floras et al. 1989; Halliwill 2001). These results suggest that post‐exercise sumpathoinhibitation may occur only after exercise training in healthy older populations.

The present study has some limitations. First, the sample size of the study this was relatively small and may have been insufficient to observe the relationship between the changes in cerebral hemodynamics and central arterial stiffness. Second, we used transcranial Doppler to measure cerebral blood flow velocity rather than cerebral blood flow. Cerebral blood flow volume such as carotid and/or vertebral artery blood flow should be investigated in the future.

## Conclusions

In conclusion, we demonstrated that a single 30‐min bout of aerobic exercise at VT intensity did not induce changes in cerebral hemodynamics such as blood flow velocity and PI in older adults; however, after a 12‐week aerobic exercise training program, an acute exercise bout of the same duration and at the same relative intensity resulted in reduced cerebral flow pulsatility. These results suggest that regular aerobic exercise may enhance the post‐exercise reduction in PI in older adults.

## Conflict of Interest

The authors declare no conflict of interest.
